# The basophil activation test reflects the severity and the threshold of allergic reactions to peanut – a double-blind-placebo-controlled peanut challenge study

**DOI:** 10.1186/2045-7022-5-S3-P25

**Published:** 2015-03-30

**Authors:** Alexandra Santos, Abdel Douiri, Alick Stephens, Suzana Radulovic, George du Toit, Victor Turcanu, Gideon Lack

**Affiliations:** 1 King's College London, London, United Kingdom

## Background

Peanut allergic patients may react to small amounts of the allergen with symptoms that can be life-threatening. The management of peanut allergy (PA) relies on allergen avoidance and adrenaline auto-injector for rescue treatment in cases at risk of anaphylaxis. Biomarkers of severity and threshold could improve the management of PA.

## Objective

To assess the utility of the basophil activation test (BAT) to predict the severity and the threshold of reactivity to peanut on double-blind-placebo-controlled-peanut-challenges (DBPCPC).

## Methods

Patients with positive DBPCPC were included in the study. The severity of the allergic reaction on DBPCPC was scored and the threshold dose was determined. Skin prick test, specific IgE to peanut and its components and BAT to peanut extract (PE) were performed on the day of the challenge.

## Results

44 peanut allergic children (median age 5 years) reacted to peanut on DBPCPC with clinical symptoms than ranged from oral allergy syndrome to anaphylaxis. 61% of patients reacted to 0.1g of peanut protein. The mean %CD63+ basophil at 10 and 100 ng/ml of PE was independently associated with severity (p=0.012) whilst CD-sens (1/EC50x100) was independently associated with threshold (p=0.039) of allergic reactions to peanut. Severity and threshold parameters were correlated both at the clinical (Rs=-0.38; p=0.013) and at the basophil level (Rs=0.65; p<0.001).

## Conclusions

Basophil reactivity and sensitivity are associated with severity and threshold of allergic reactions to peanut on DBPCPC. Further studies are needed to define prognostic cut-offs values for BAT to determine severity and thresholds of reactivity in peanut allergic patients.

